# Diversity of salt tolerance in *Vigna nakashimae*, wild related species of the azuki bean (*Vigna angularis*)

**DOI:** 10.1270/jsbbs.23050

**Published:** 2024-03-29

**Authors:** Eri Ogiso-Tanaka, Sompong Chankaew, Takehisa Isemura, Rusama Marubodee, Alisa Kongjaimun, Akiko Baba-Kasai, Kazutoshi Okuno, Hiroshi Ehara, Norihiko Tomooka

**Affiliations:** 1 Genetic Resources Center, National Institute of Agrobiological Sciences (NIAS), 2-1-2 Kannondai, Tsukuba, Ibaraki 305-8602, Japan; 2 Program in Plant Breeding, Faculty of Agriculture at Kamphaeng Saen, Kasetsart University, Kamphaeng Saen, Nakhon Pathom 73140, Thailand; 3 Graduate School of Bioresources, Mie University, 1577 Kurimamachiya-cho, Tsu, Mie 514-0102, Japan; 4 Graduate School of Life and Environmental Sciences, University of Tsukuba, 1-1-1 Tennodai, Tsukuba, Ibaraki 305-8571, Japan

**Keywords:** *Vigna nakashimae*, Azuki bean, *Vigna angularis*, salt tolerance, genetic resource, MIGseq, RADseq

## Abstract

*Vigna nakashimae* is a wild species closely related to the azuki bean (*V. angularis*), with salt-tolerance abilities. The present study aimed to explore the genetic and salt tolerance diversity within the species, by evaluating the phylogenetic relationships of 55 accessions of *V. nakashimae* including 25 newly collected from the Gotō Islands and Iki in Nagasaki Prefecture, Japan. We conducted salt-tolerance analysis for 48 of the accessions, including 18 of the newly collected accessions. Phylogenetic analysis based on single-nucleotide polymorphisms obtained from MIGseq and RADseq analyses revealed the genetic diversity of *V. nakashimae* to reflect the geographic arrangement of the habitat islands. Korean accessions formed one clade, while Japanese accessions predominantly grouped into Uku Island and Fukue Island subclades. Within this population, we identified “G4-2” (JP248291) as the most salt tolerant, surpassing even the previously reported “Ukushima” accession. Both accessions collected from Uku Island, with accessions belonging to the Uku Island subclade exhibiting a strong trend of salt tolerance. Our results strongly suggest the occurrence of genetic mutations conferring enhanced salt tolerance in specific clade and region. This study highlights the potential of genetic analyses for identifying regions suitable for collecting valuable genetic resources for stress tolerance.

## Introduction

Azuki bean (*Vigna angularis*) is a species of the subgenus *Vigna* which is a food legume for people in East and South Asia, particularly Japan, China, Korea, Nepal and Bhutan (Vaughan *et al.* 2004). The bean is a source of dietary protein for these regional consumers, where it is typically cooked with rice or used as an ingredient in sweet confectioneries. In Japan, azuki beans are the second most important legume crop, after soybeans, where they are primarily used to make a paste for sweets or consumed as sugar-glazed boiled beans. More than 90% of the azuki bean produced in Japan are cultivated in Hokkaido, an environment optimized for production (MAFF 2022). In contrast, some of the crop production regions outside of Japan, they are affected by coastal conditions or underground salt rock. It has been reported that 20%–50% of the world’s irrigated cropland consists of soil with salinity conditions (FAO 2021). Salinity is a limiting factor for azuki bean production and productivity, and cause plant death. Azuki bean exhibited the lowest level of salt tolerance among *Vigna* species and will rapidly wilted under even mild salt stress (Yoshida *et al.* 2016). Hence, the introduction of salt-tolerance genes from cross compatible wild relatives of the azuki bean is a promising genetic approach for improving salt tolerance of azuki bean plants.

Based on the widely varied habitats of wild *Vigna* species (as described by Tomooka *et al.* 2011), a halophytic *Vigna* species—beach cowpea (*V. marina* subsp. *oblonga*)—has previously been investigated for salt tolerance using QTL analysis (Chankaew *et al.* 2014). Six further *Vigna* species; namely, *V. marina*, *V. luteola*, *V. trilobata*, *V. vexillata*, *V. nakashimae*, and *V. riukiuensis* exhibit salt tolerance up to 200 mM (NaCl) (Iseki *et al.* 2016, Yoshida *et al.* 2020). *V. nakashimae* and *V. riukiuensis* are cross compatible with the azuki bean and have different mechanisms of salt tolerance (Noda *et al.* 2022, Ogiso-Tanaka *et al.* 2023, Yoshida *et al.* 2016). *V. nakashimae* grows in South Korea, northeastern China, Kyushu (Japan), and neighboring remote islands (Tomooka *et al.* 2013). By screening salt tolerance of 31 accessions of *V. nakashimae* (30 from Korea and one from Japan) under soil-cultured conditions, Yoshida *et al.* (2016) identified the most salt tolerant accession JP212341 from Korea. They simultaneously evaluated salt tolerance under hydroponic cultivation conditions using five accessions of *V. nakashimae*, and found “Ukushima (JP107879)” to exhibit the highest salt tolerance (Yoshida *et al.* 2016). At that time, only one accession of *V. nakashimae* from Japan was recorded at National Agriculture and Food Research Organization (NARO) Genebank in Japan; thus, Tomooka *et al.* (2013) collected an additional 24 accessions of this species from the Gotō Island and Iki Island in Nagasaki prefecture, Japan. The present study aimed to gain insight into the genetic background underlying salt tolerance by analyzing the genetic diversity of *V. nakashimae* accessions in the NARO Genebank, including the newly collected accessions, using single-nucleotide polymorphisms (SNPs) derived from multiplexed inter-simple sequence repeat genotyping (MIGseq) and restriction site-associated DNA sequencing (RADseq).

## Materials and Methods

### Plant materials

**Fig. 1.**
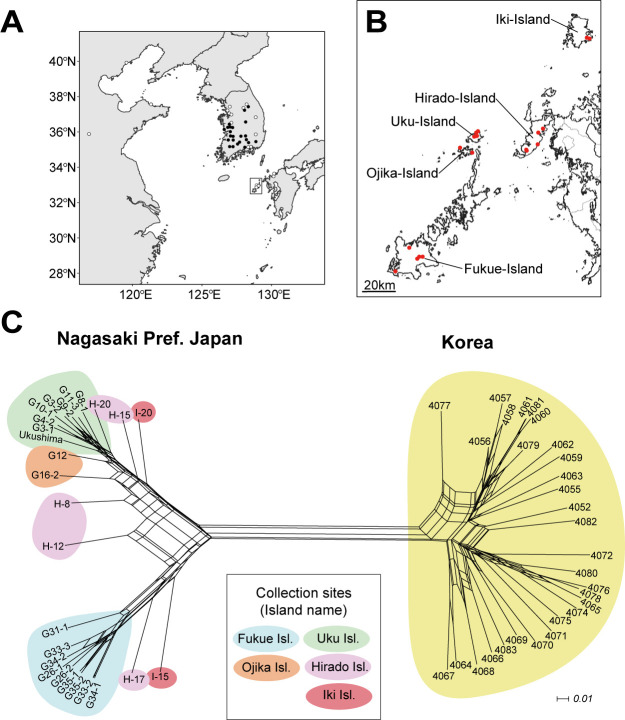
Geographic distribution of *Vigna nakashimae* accessions based on direct collection. (A) Map showing the entire distribution range of the species. White circles indicate collection sites of herbarium specimens, black circles indicate direct-collection sites for gene bank samples. (B) Map showing direct-collection areas for the present study in the Gotō Islands, Nagasaki Prefecture, Japan. (C) Phylogenetic network was created using the split-tree program (Huson and Bryant 2006) with the final set of 4,604 SNPs.

The 55 accessions of *V. nakashimae* maintained in the NARO Genebank were used in the present study (Table 1, Fig. 1A, 1B). Among them, 29 were from Korea and one was from Uku Island in the northernmost of the Gotō Islands in Nagasaki Pref., Japan (Yoshida *et al.* 2016). The remaining 25 accessions were newly collected from the Gotō-Islands and Iki Island in 2012, as reported by Tomooka *et al.* (2013) and Takahashi *et al.* (2014). All Korean accessions and Ukushima were purified using the single-seed-descent method (Table 1). The 29 accessions from Korea underwent self-pollination for more than four generations since their collection, while the other 18 (Tomooka *et al.* 2013) and 7 accessions (Takahashi *et al.* 2014) underwent two or one generations of self-pollination since their collection in 2012 or 2013 (Table 1).

### Growth conditions and Evaluation of salt tolerance

Seeds were sown in trays with Seramis clay granules (SERAMIS^®^), supported, and kept in a growth chamber at 25°C for 10 days. Each accession was transplanted to hydroponic culture in a greenhouse at the National Institute of Agrobiological Sciences (NIAS; 36°03ʹN, 140°10ʹE) and cultivated under short-day conditions (10 hr light/14 hr dark) from April 2013 to June 2014. The culture contained a diluted nutrient solution of 1:1 Otsuka house 1.5 g/l:Otsuka house 1 g/l (Otsuka Chemical Co., Osaka, Japan: N, P, K, Ca, and Mg = 18.6, 5.1, 8.6, 8.2, and 3.0 mEq/l, respectively). The final solution was adjusted to an electrolytic conductivity of 100 mS/m with water. Three weeks after transplanting, NaCl was added to the nutrient solution at 4-day intervals at a starting concentration of 50 mM, increasing in 50 mM steps to a final concentration of 200 mM. The NaCl concentration was then maintained at 200 mM for 2 weeks (Chankaew *et al.* 2014).

Percentage score of wilted leaves (PWL) was recorded for each accession by visual scoring using four categories: 1 = 0%–15% leaves had wilted, 3 = 16%–35% leaves had wilted, 5 = 35%–65% leaves had wilted, 7 = 65%–85% leaves had wilted, and 9 = >85% leaves had wilted or the plant died completely. The PWL scores were recorded at 5 and 9 days after the NaCl concentration reached 200 mM. The salt tolerance of three plants were evaluated through one repetition, and this process was repeated three times.

### Genetic diversity analysis using MIGseq and RADseq

We extracted DNA from all samples using the modified cetyltrimethylammonium bromide (CTAB) method (Lodhi *et al.* 1994). The MIGseq and RADseq libraries were prepared as described in Suyama and Matsuki (2015) and Marubodee *et al.* (2015), respectively. MIGseq and RADseq were performed using the same DNA samples (Iki-15 only has RADseq data). The DNA libraries were sequenced using an Illumina MiSeq Sequencer with MiSeq Reagent Kit V3, run for 150 cycles (Illumina, CA, USA) for MIGseq and a HiSeq2000 (Illumina) for RADseq, respectively. Therefore, we obtained paired-end sequences of 80 bp from read 1 and 94 bp from read 2 from MiSeq for MIGseq, single-end sequences of 101 bp from HiSeq 2000 for RADseq, respectively. First, sequencing adaptors and low-quality bases were removed via Trimmomatic-0.33 (Bolger *et al.* 2014). The trimmed reads were mapped onto the *V. nakashimae* JP247291 reference genome (https://viggs.dna.affrc.go.jp/download_Vnakashimae_v1, G418[G4-2] in Ogiso-Tanaka *et al.* 2020) using BWA 0.7.17-r1188 (Li and Durbin 2009). Subsequently, BAM files obtained from MIGseq and RADseq were merged and sorted, and SNP calling was conducted using reference-based analysis pipeline of STACKS 2 (Rochette *et al.* 2019). Moreover, SNPs were filtered using vcftools (Danecek *et al.* 2011) with the following parameters: (1) minimum sequence depth was above 5, (2) minor allele frequency was greater than 0.05, and (3) missing rate was lower than 10%. Output VCF file was converted to the NEXUS format from VCF file using PGDSpider v.2.1.15 (Lischer *et al.* 2014). In order to reconstruct possible network-like evolutionary relationships among species, SplitsTree4 software (Huson and Bryant 2006) was used to generate the split-network by implementing neighbor net analysis with variance of ordinary least squared. PCA was conducted using PLINK v1.90b7.1 (Purcell *et al.* 2007) and filtered SNP data, which were analyzed using STRUCTURE v2.3.4 with the admixture model to estimate population genetic structures (Pritchard *et al.* 2000). Genetic diversity analysis was conducted using *poppr* with R 4.3.2 (Kamvar *et al.* 2014) and GENODIVE (Meirmans 2020). Sequence data were deposited in the Sequence Read Archive under the bioproject accession number PRJBD11715 (DRA012087).

## Results and Discussion

We obtained 2.5 Gb of data containing 12,928,581 pairs of raw reads, which equated to 11,465,210 clean reads after filtering with an average of 208,458 ± 9,953 reads (mean ± standard error [SE]) per sample from MIGseq. From RADseq analysis, we obtained 16.1 Gb of data containing 187,617,514 clean reads after filtering, with an average of 3,411,228 ± 127,406 reads (mean ± SE) per sample. We aligned MIGseq and RADseq data to reference genome, combined them and performed SNP calling. We genotyped 33,951 variant sites on 471,934 loci. After filtering, a total of 4,604 variant sites (average coverage of 59.9x, range: 9.7–89.7x, stdev: 16.8x) for each locus from 55 accessions were used for all analysis.

We performed split-network analysis using the SplitsTree program (Huson and Bryant 2006). The creation of a phylogenetic network based on concatenated SNP sites revealed two large groups related to Korea and Japan, which contained subclades corresponding to individual islands of Japan (Fig. 1C). The analysis revealed that the Japan subclade diverged into two main subclades. The first subclade comprised all accessions from Fukue Island and two accessions from Hirado Island and Iki Island. The second subclade was mostly composed of all accessions from Uku and Ojika Island, as well as some accessions from Hirado (two accessions) and Iki (one accession) Island. The two accessions from Hirado Island were in an intermediate position between these two subclades (Fig. 1C). In contrast, clear branching patterns were not observed in the accessions from Korea. These results were similar in the case of analyzing RADseq or MIGseq alone (Supplemental Text 1, Supplemental Fig. 1). However, some accessions from Hirado Island were placed into different clades by MIGseq and RADseq (Supplemental Fig. 1). Upon examining the regions of SNPs used for analysis, no significant differences were observed in their distribution on chromosomes between MIGseq and RADseq (Supplemental Fig. 2). Therefore, the reason for the variation in clade placement between the methods is likely attributed to the differences in sequencing data quantity, SNP numbers, or sequencing strategies: MIGseq sequenced around simple sequence repeat regions, whereas RADseq sequenced around restriction enzyme sites. These results suggest the independent evolution of *V. nakashimae* on the Uku Island and Fukue Islands, which are separated by a mere 70 km. Regarding the two accessions from Iki Island, despite their collection sites being close, one belonged to the subclades of Uku Island, while the other belonged to subclade of Fukue Island. This suggests that seeds transported in past from Fukue Island and Uku Island by some factors, such as ocean currents, have been maintained without genetic admixture. By adding new collections from the Gotō Islands, *V. nakashimae* has genetically diversified within this geographically constrained region.

**Fig. 2.**
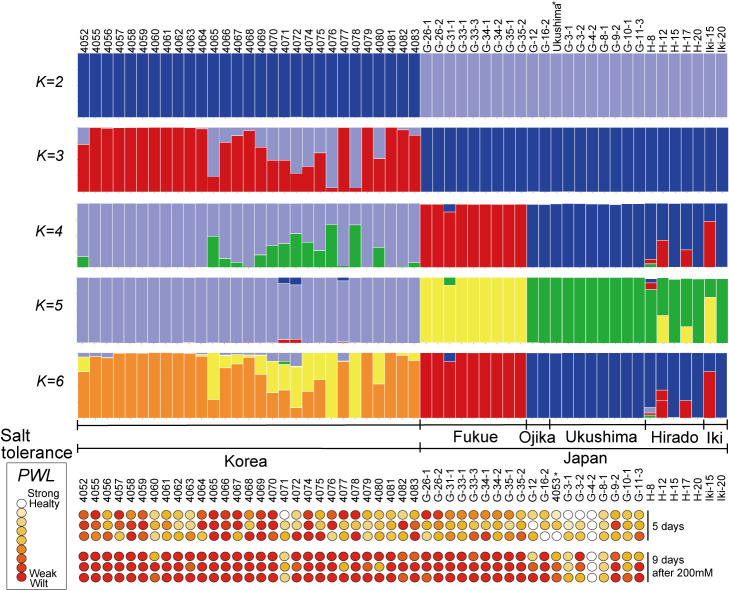
Illustration of the genetic structure of *Vigna nakashimae* and salt tolerance. (Upper) Bar plot showing the estimated population structure at *K* = 2–6 in 55 accessions from MIGseq and RADseq merged data. (Lower) Heat maps of the percent score of wilted leaves in hydroponic culture with 200 mM NaCl. A score of 1 (no damage) is represented with white circles, a score of 9 (dead) is indicated with red circles. One circle represents a single repetition of the salt tolerant test. The number of days after 200 mM NaCl was reached is indicated on the right side. Abbreviations: PWL, percentage score of wilt leaves.

To elucidate the geographic structuring of genetic diversity, relationship between genetic structures among 55 accessions was evaluated (Fig. 2). At *K* = 2, Korea and Japan groups were divided into two clusters. At *K* = 3, Japan formed a single cluster, whereas Korea exhibited a continuous pattern. For *K* = 4 or higher, Japan was divided into two clusters but there was no distinct cluster within the Korean samples. At *K* = 5, populations from Korea, Fukue Island, and Uku Island were divided into three genetic clusters, corresponding most closely to geographical distribution. Hirado and Iki Islands showed some degree of intermixing at *K* = 4–6. The PCA scatterplot also indicated that accessions from Korea and Japan were distributed into two genetic clusters (Supplemental Fig. 3A), similar to the results of the phylogenetic network and STRUCTURE analyses (Figs. 1, 2). In the PCA plots (Supplemental Fig. 3A), accessions of Korea and Japan separated along PC1, while those of Fukue and Uku Island in Japan were separated in PC2. Korea forms a single cluster along PC1 and PC2 and exhibits a continuous distribution along PC3. The PCA using only the Korean dataset also indicated a continuous distribution (Supplemental Fig. 4A). This is likely due to the absence of significant geographical barriers, such as seas or major rivers, suggesting the absence of genetic isolation. The results of PCA using only the Japanese dataset revealed the genetic differentiation among Japanese accessions (Supplemental Fig. 3B), which showed Fukue Island to be clearly diverged from Uku Island along PC1. Hirado Island displayed greater diversity than other populations, with some Hirado Island accessions placing closer to Uku Island than Fukue Island, reflecting the geographic relationships. Similar trends were observed when analyzing MIGseq and RADseq separately (Supplemental Fig. 5A, 5B). The diversity indices showed an expected heterozygosity (*He*) of 0.1–0.354, with Korea at 0.241 and Japan at 0.229, indicating moderate diversity in both populations (Supplemental Table 1). As these values were compared to populations from across Korea as a whole and a very limited area around the Gotō Islands in Japan, it suggests that *V. nakashimae* in Japan has diversified in the narrow region. The Shannon diversity index values also showed a similar trend, with Korea at 1.462 and Japan at 1.415 (Supplemental Table 1). This genetic diversity within the Gotō Islands, including Uku Island where the accession with the highest salt tolerance was found, which suggests the possibility of salt tolerance diversity within this area.

We evaluated the salt tolerance of 48 accessions with sufficient seed availability in hydroponic culture. The PWL values were visualized as a heatmap (Fig. 2). Most accessions exhibited severe damage upon salt exposure, with approximately a quarter of the samples observed to be wilted after 5 days of exposure to 200 mM NaCl, and nearly 4/5 wilted after 9 days of exposure. Comparing these results with those of Yoshida *et al.* (2016), differences in the strength of salt tolerance among the Korean accessions were observed. However, Ukushima, which exhibited the highest salt tolerance among the accessions used in their study, was consistently replicated, confirming the effectiveness of hydroponic cultivation in evaluating salt tolerance. The newly collected G4-2 (JP247291) from Uku Island also have the highest salt tolerance (average PWL = 1.0), with hardly any observable wilting even after 9 days of cultivation with 200 mM NaCl. In addition, the G3-1, 4071, G8-1, and Ukushima (4053) accessions exhibited strong salt tolerance with varying degrees of wilting from 30% to 50% observed (average PWL = 3.0, 3.5, 4.2, and 4.3, respectively) (Fig. 2).

**Fig. 3.**
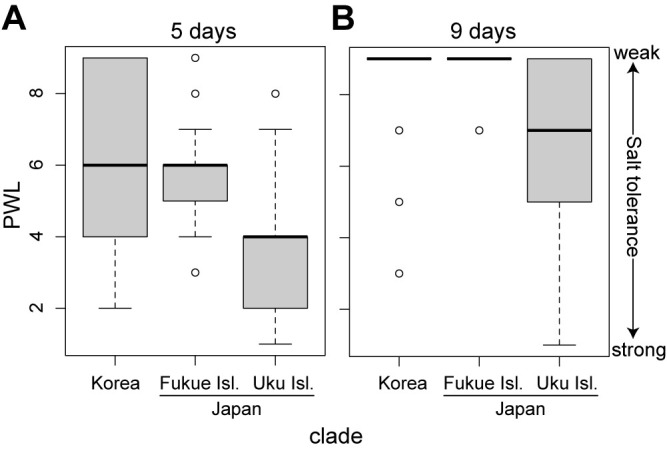
Box and whisker graphs showing the degree of salt tolerance within each separate clade in the phylogenetic network. (A) 5 days and (B) 9 days after 200 mM salt treatment. Abbreviations: PWL, percentage score of wilted leaves.

When comparing salt tolerance and phylogenetic relationships, all of the Japan accessions that exhibited strong salt tolerance originated from Uku Island and were categorized into one subclade (Figs. 1–3). We determined the two most salt-tolerant accessions, G4-2 and G3-1, to be very closely genetically related to Ukushima (Fig. 1C). This suggests that the salt tolerance observed for G4-2, G3-1, G8-1, and Ukushima might have evolved within this clade. The habitat of these salt tolerant accessions are seaside grasslands with strong sea winds, possibly acting as a selection pressure for salt tolerance. Although Fukue Island is located not far from Uku Island within Gotō Islands, the accessions of Fukue Island were genetically differentiated and showed low levels of salt tolerance (Figs. 1–3). The habitat of salt susceptible accessions from Fukue Island are inland paddy field area without strong sea wind. All the Korean accessions were genetically differentiated from Japan accessions and showed low levels of salt tolerance (except for 4071) (Figs. 2, 3). Among them, 11 Korean accessions had very low salt tolerance (average PWL scores of 8 or higher). The Korean accession 4065 (JP254467) exhibited particularly weak salt tolerance, with all individual plants dying completely after 5 days of treatment with 200 mM NaCl. Interestingly, only one Korean accession 4071 showed high level of salt tolerance. While in Yoshida *et al.* (2016), this accession showed the highest salt tolerance under soil cultivation condition, it was collected from the paddy field area located at the central park of Korea far from sea (Supplemental Fig. 4B). This level of high salt tolerance was not observed in other closely genetically related accessions from Korea. These facts imply a distinct origin of salt tolerance for accessions from Uku Island and for Korean accession 4071, suggesting that different genes or mechanisms might underlie specific salt-tolerance traits.

This study indicates the effectiveness of phylogenetic/STRUCTURE analysis of data from genetic resources for identifying genetic clade(s) with strong phenotypes. Using phylogenetic relationships to preemptively predict regions where populations with stress tolerance are growing based on phylogenetic relationships might enable us to efficiently select collection sites and collect individuals with high tolerance. Furthermore, the discovery of private mutations, like accession 4071 in the present study, highlights the importance of diligently expanding collections and screening for traits such as stress tolerance.

## Author Contribution Statement

Conceptualization: E.O.T., S.C., and N.T.; methodology: E.O.T. and S.C.; validation: S.C.; formal analysis: E.O.T.; investigation: E.O.T., S.C., T.I., R.M., A.K., and A.B.; data curation: E.O.T.; writing—original draft preparation: E.O.T. and S.C.; writing—review and editing: E.O.T., S.C., K.O., and N.T.; supervision: N.T.; project administration: E.O.T.; funding acquisition: E.O.T. and N.T. All authors have read and agreed to the published version of the manuscript.

## Supplementary Material

Supplemental Figures

Supplemental Table

Supplemental Text

## Figures and Tables

**Table 1.** Accessions of *Vigna nakashimae* used in this study

Strain ID*^a^*	JP number*^b^*	Original JP number*^c^*	Collection site	Origin	SSD generation*^d^*
4052	JP254454	JP81231	Hyanggyo-dong, Namwon-si	Korea	6
4055	JP254457	JP212325	Hongcheon, Kangweon-do.	Korea	5
4056	JP254458	JP212326	Suwon, Gyeonggi-do.	Korea	6
4057	JP254459	JP212327	Yeoju. Gyeonggi-do.	Korea	6
4058	JP254460	JP212328	Yeoju, Gyeonggi-do.	Korea	6
4059	JP254461	JP212329	Incheon.	Korea	6
4060	JP254462	JP212330	Pyeongtaek. Gyeonggi-do.	Korea	5
4061	JP254463	JP212331	Hwaseong. Gyeonggi-do.	Korea	5
4062	JP254464	JP212332	Hwaseong. Gyeonggi-do.	Korea	5
4063	JP254465	JP212333	Hwaseong, Gyeonggi-do.	Korea	5
4064	JP254466	JP212334	Geochang, Gyeongsangnam-do.	Korea	4
4065	JP254467	JP212335	Geochang, Gyeongsangnam-do.	Korea	4
4066	JP254468	JP212336	Changnyeong, Gyeongsangnam-do.	Korea	4
4067	JP254469	JP212337	Hamyang, Gyeongsangnam-do.	Korea	6
4068	JP254470	JP212338	Goryeong, Gyeongsangbuk-do.	Korea	4
4069	JP254471	JP212339	Geumreung, Kyeongbuck.	Korea	4
4070	JP254472	JP212340	Namwon, Jeollabuk-do.	Korea	4
4071	JP254473	JP212341	Muju, Jeollabuk-do.	Korea	6
4074	JP254476	JP212344	Dangjin, Chungcheongnam-do.	Korea	4
4075	JP254477	JP212345	Daejeon.	Korea	6
4076	JP254478	JP212346	Buyeo, Chungcheongnam-do.	Korea	6
4077	JP254479	JP212347	Buyeo, Chungcheongnam.	Korea	6
4078	JP254480	JP212348	Buyeo, Chungcheongnam-do.	Korea	6
4079	JP254481	JP212349	Yesan, Chungcheongnam-do.	Korea	6
4080	JP254482	JP212350	Cheongyang, Chungcheongnam-do.	Korea	6
4081	JP254483	JP212351	Taean, Chungcheongnam-do.	Korea	5
4082	JP254484	JP212352	Goesan, Chungcheongbuk-do.	Korea	6
4083	JP254485	JP212353	Gaesan. Chungbuk.	Korea	5
4053 “Ukushima”*^e^*	JP254455	JP107879	Uku Island	Nagasaki, Japan	5
G-3-1	JP247288		Uku Island	Nagasaki, Japan	2~3
G-3-2	JP247289		Uku Island	Nagasaki, Japan	2~3
G-4-2	JP247291		Uku Island	Nagasaki, Japan	2~3
G-8-1	JP247300		Uku Island	Nagasaki, Japan	2~3
G-9-2	JP247303		Uku Island	Nagasaki, Japan	2~3
G-10-1	JP247304		Uku Island	Nagasaki, Japan	2~3
G-11-3	JP247309		Uku Island	Nagasaki, Japan	2~3
G-12	JP247313		Ojika Island	Nagasaki, Japan	2~3
G-16-2	JP247321		Ojika Island	Nagasaki, Japan	2~3
G-26-1	JP247341		Fukue Island	Nagasaki, Japan	2~3
G-26-2	JP247342		Fukue Island	Nagasaki, Japan	2~3
G-31-3	JP247358		Fukue Island	Nagasaki, Japan	2~3
G-33-2	JP247361		Fukue Island	Nagasaki, Japan	2~3
G-33-3	JP247362		Fukue Island	Nagasaki, Japan	2~3
G-34-1	JP247363		Fukue Island	Nagasaki, Japan	2~3
G-34-2	JP247364		Fukue Island	Nagasaki, Japan	2~3
G-35-1	JP247365		Fukue Island	Nagasaki, Japan	2~3
G-35-2	JP247366		Fukue Island	Nagasaki, Japan	2~3
H-8	JP251218		Hirado Island	Nagasaki, Japan	1
H-12	JP251222		Hirado Island	Nagasaki, Japan	1
H-15	JP251225		Hirado Island	Nagasaki, Japan	1
H-17	JP251228		Hirado Island	Nagasaki, Japan	1
H-20	JP251231		Hirado Island	Nagasaki, Japan	1
I-15	JP255947		Iki Island	Nagasaki, Japan	1
I-20	JP255948		Iki Island	Nagasaki, Japan	1

*^a^* ID numbers represent the accession name used in this study. *^b^* JP numbers were assigned in the present study. The Korean accessions and Ukushima were derived from a lineage propagated from a single-seed descent from the original JP number. *^c^* The original JP number represents the self-pollinated parent of single-seed descent lines. The underlined accessions underwent salt-tolerance screening under hydroponic culture conditions in the experiments of Yoshida *et al.* (2016). *^d^* Single-seed descent generation indicates the generation at the time of the salt-tolerance test. *^e^* The strain ID 4053 is the accession “Ukushima”, which has been identified as the most salt-tolerant strain of *Vigna nakashimae* by Yoshida *et al.* (2016), and has been used in QTL analysis by Ogiso-Tanaka *et al.* (2023).
