# Supplementary material for: Diversity of salt tolerance in Vigna nakashimae, wild related species of the azuki bean (Vigna angularis)
Source: Breed Sci. 2024 Mar 29;74(2):166–72. doi: 10.1270/jsbbs.23050 (PMC11442110; doi:10.1270/jsbbs.23050)
Supplement: Supplementary file 1 — Supplemental Figures [file 74_166_s1.pdf]

doi: 10.1270/jsbbs.23050

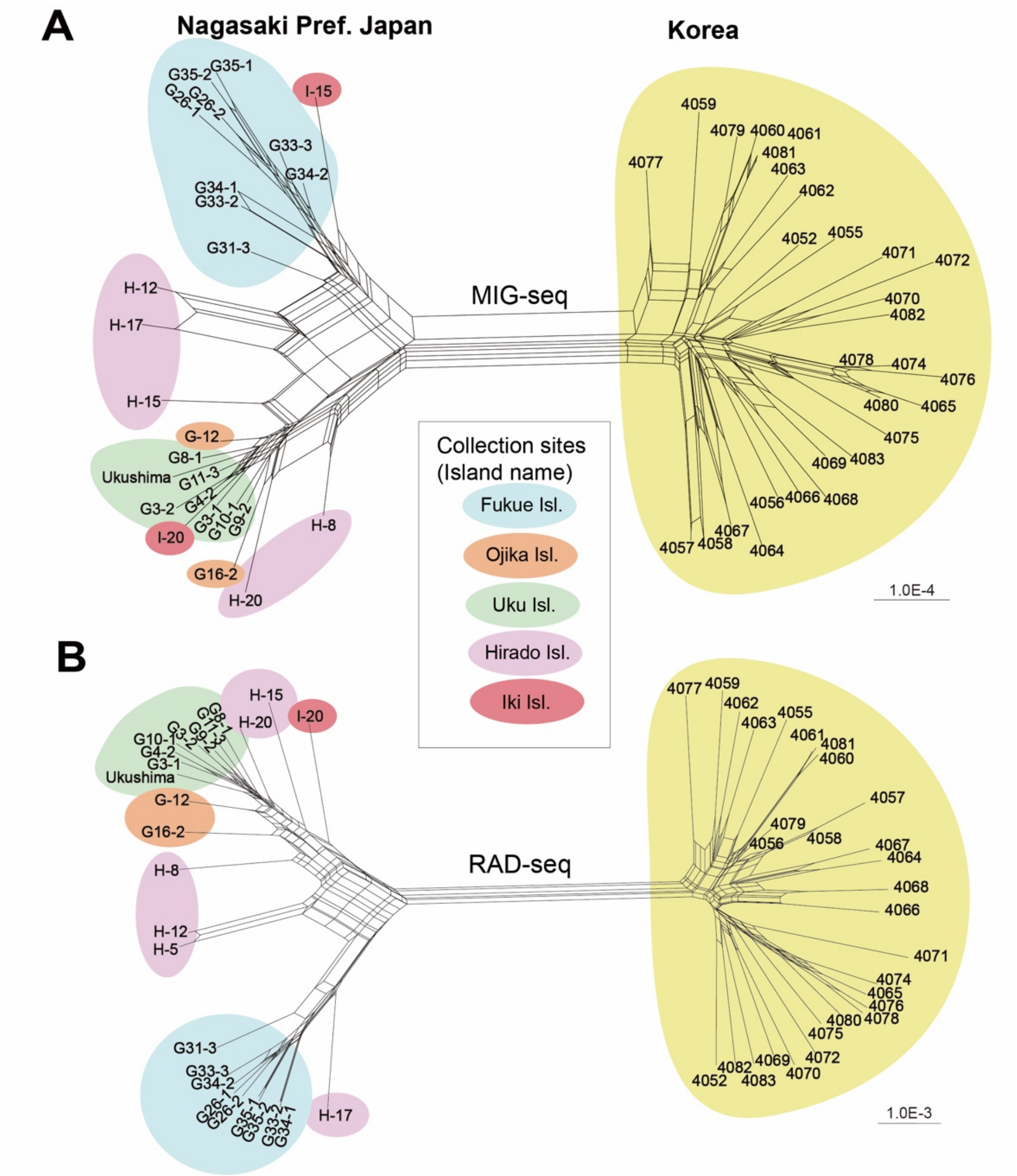

Supplemental Figure 1

Phylogenetic network based on SNPs derived from *de novo* analysis using iPyrad. Split-network analysis was performed using the split-tree program (Huson and Bryant 2006) with a dataset of 1,299 SNPs from MIGseq and 12,412 SNPs from RADseq.

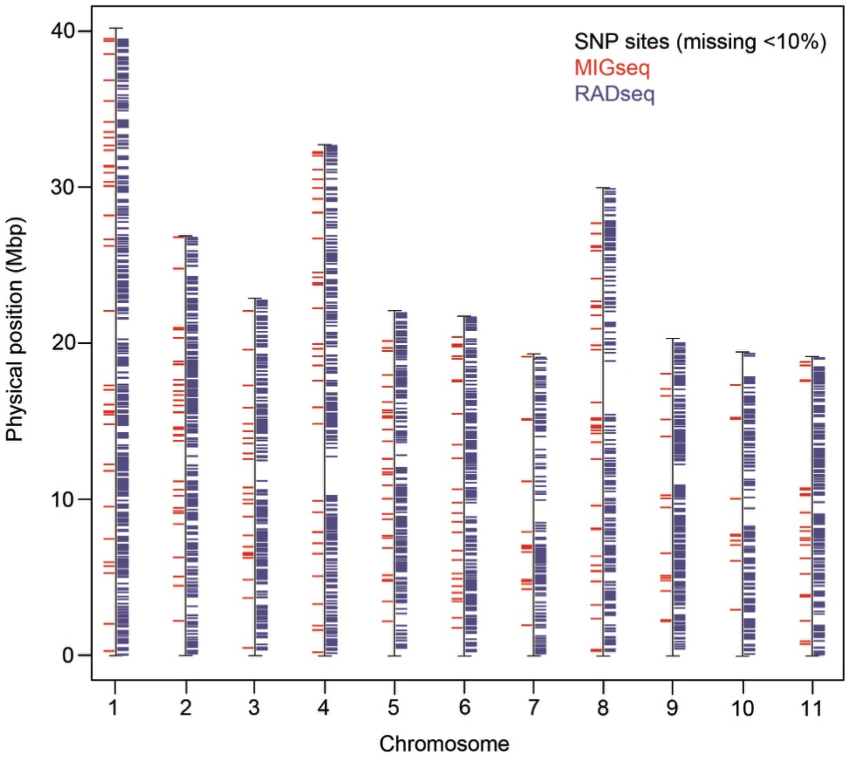

Supplemental Figure 2

Distributions of SNP sites by MIGseq (543 sites) and RADseq (4,242 sites) are represented by relative physical positions on *V. nakashimae* chromosomes. This indicates only the regions where genotyping was possible in over 90% of the samples (missing < 0.1, depth > 5, MAF > 0.05). Each red color indicates MIGseq (left), while purple (right) indicates RADseq. No regions could be genotyped in the other four contigs.

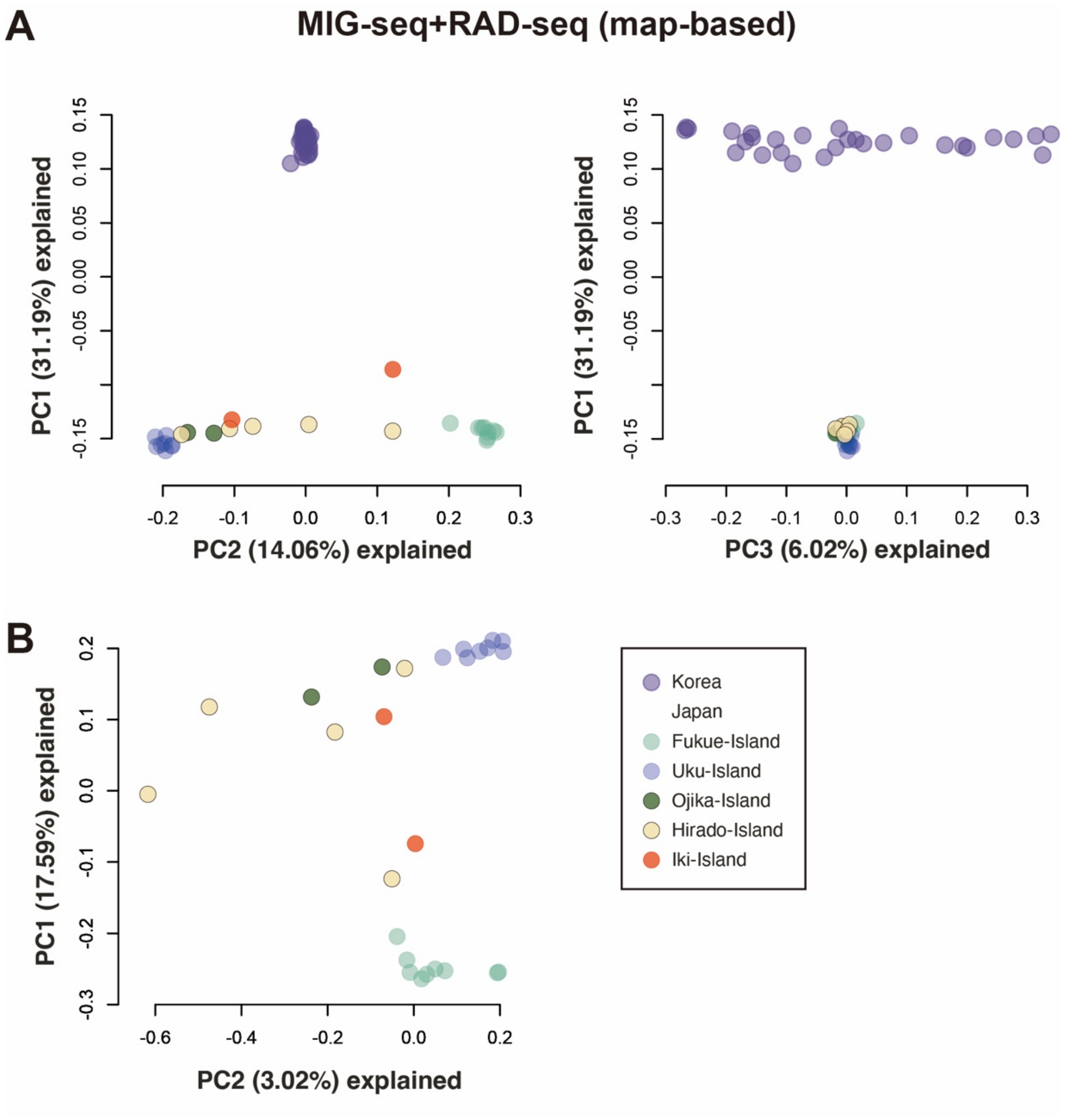

Supplemental Figure 3

Scatterplots showing individual variation based on principal component analysis scores from the reference genome map-based analysis of merged data both multiplexed inter-simple sequence repeats genotyping by sequencing and restriction-site-associated DNA marker sequencing data. (A) Scatterplots showing the results of all 55 accessions (4,604 SNPs). (B) Scatterplots showing the results of all 29 accessions derived from Japan (2,601 SNPs). Abbreviations: MIGseq, multiplexed inter-simple sequence repeats genotyping by sequencing; PCA, principal component analysis; RADseq, restriction-site-associated DNA marker sequencing

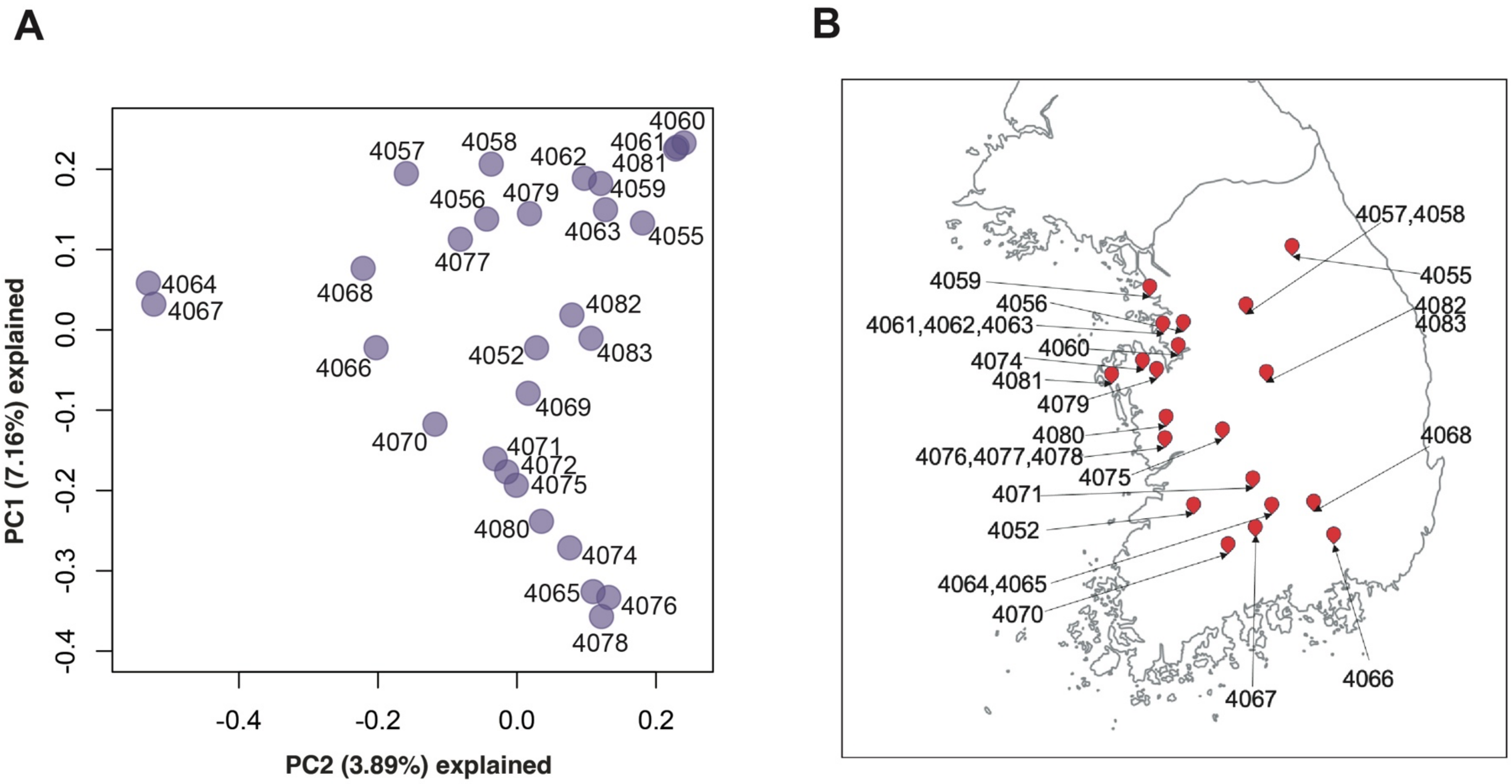

Supplemental Figure 4

Collection locations of *V. nakashimae* distribution in Korea and PCA scatterplots. PCA showing individual variation based on PCA scores from the reference genome map-based analysis of merged data from both MIGseq and RADseq of Korean accessions (3,400 SNPs).

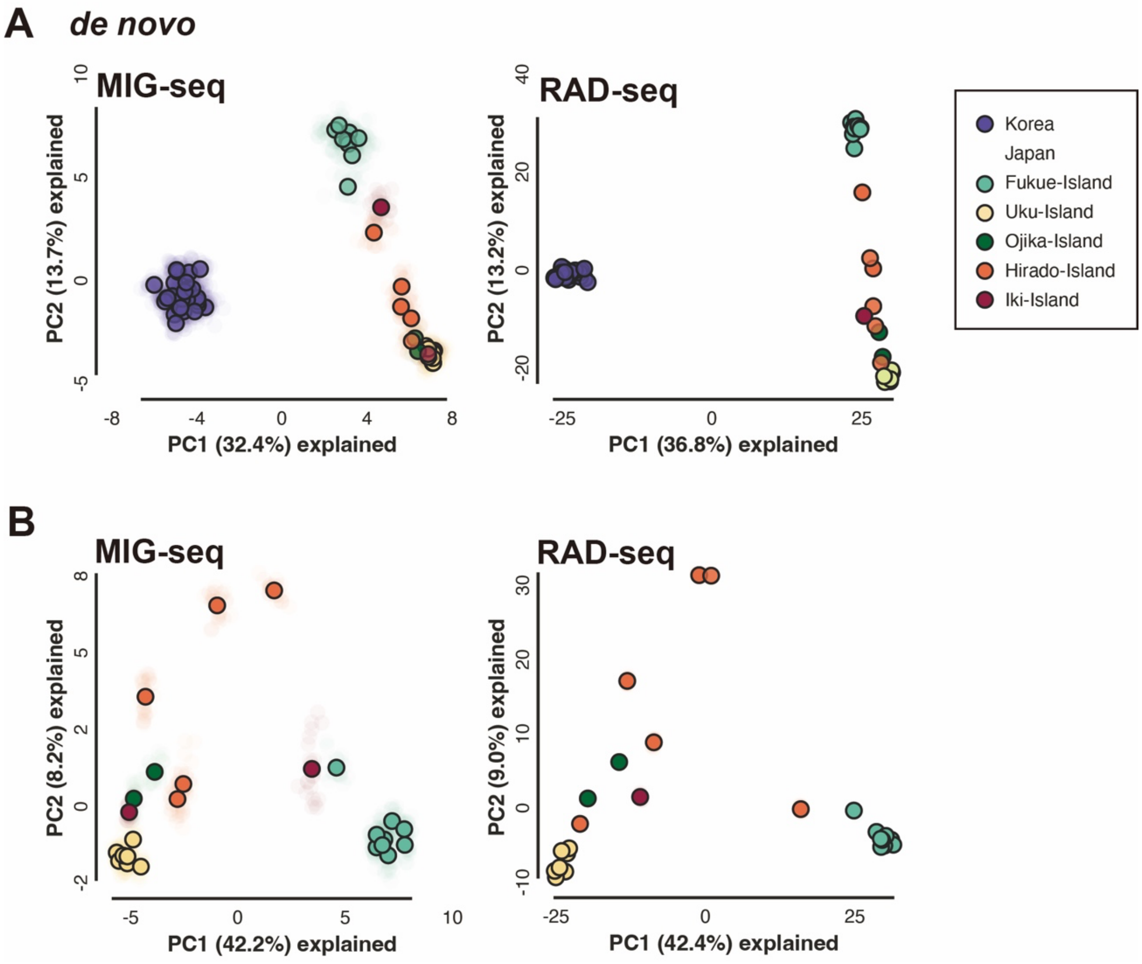

Supplemental Figure 5

Scatterplots showing individual variation based on *de novo* analysis in principal component analysis scores from 25 replicates derived from MIGseq and RADseq. (A) Scatterplots showing the results of all 55 (MIGseq: 1,299 SNPs) and 54 (RADseq: 12,412 SNPs) accessions. (B) Scatterplots showing the results of all 29 (MIGseq: 916 SNPs) and 29 accessions (RADseq: 4,864 SNPs) derived from Japan.
