# Supplementary material for: Diversity of salt tolerance in Vigna nakashimae, wild related species of the azuki bean (Vigna angularis)
Source: Breed Sci. 2024 Mar 29;74(2):166–72. doi: 10.1270/jsbbs.23050 (PMC11442110; doi:10.1270/jsbbs.23050)
Supplement: Supplementary file 2 — Supplemental Table [file 74_166_s2.pdf]

doi: 10.1270/jsbbs.23050

Supplemental Table 1

Genetic variation among populations on diversity of 55 *V. nakashimae* accessions.

Populations (Origin of accessions)	Nunber of accessions	Na	Ne	Ho	He	G_{is}	H'
Korea	29	1.698	1.405	0.114	0.241	0.528	1.462
Japan	26	1.603	1.391	0.075	0.229	0.672	1.415
Fukue Island	9	1.288	1.207	0.087	0.124	0.301	0.954
Ojika Island	2	1.168	1.162	0.068	0.130	0.477	0.301
Uku Island	8	1.225	1.159	0.063	0.097	0.350	0.903
Hirado Island	5	1.445	1.298	0.077	0.208	0.631	0.699
Iki Island	2	1.543	1.536	0.370	0.354	-0.047	0.301

*Na, observed number of alleles

Ne, effective number of alleles

Ho, observed heterozygosity

He, expected heterozygosity

G_{is}, Inbreeding Coefficient

H', Shannon-Wiener index
