# Supplementary material for: Diversity of salt tolerance in Vigna nakashimae, wild related species of the azuki bean (Vigna angularis)
Source: Breed Sci. 2024 Mar 29;74(2):166–72. doi: 10.1270/jsbbs.23050 (PMC11442110; doi:10.1270/jsbbs.23050)
Supplement: Supplementary file 3 — Supplemental Text [file 74_166_s3.pdf]

doi: 10.1270/jsbbs.23050

Supplemental Text 1.

Genetic diversity analysis using MIGseq and RADseq map-based SNP data

Genetic diversity analysis was conducted using *poppr* with R 4.3.2 (Kamvar *et al.* 2014) and GENODIVE (Meirmans 2020) using 4,604 SNPs. The observed number of alleles (Na), effective number of alleles (Ne), observed heterozygosity (Ho), expected heterozygosity (He), Inbreeding Coefficient (G_{is}), and Shannon-Wiener index (Shi) were calculated. Principal component analysis (PCA) was performed using PLINK (Purcell *et al.* 2007).

De novo SNP detection and Genetic diversity analysis using MIGseq and RADseq data

The same DNA was used for MIGseq and RADseq, with only MIGseq data available for Iki-15. In both methods, reads were assembled, and SNPs were obtained using ipyrad 0.9.78 (Eaton and Overcast 2020). PCA analysis was also conducted (Eaton and Overcast 2020, Evanno *et al.* 2005), setting the clustering threshold between 0.85 and 0.95. Since results showed no significant variation within this range, we adopted the 0.95 threshold. For SNP data, the reconstruct potential network-like evolutionary relationships among species, we utilized SplitsTree4 software (Huson and Bryant 2006), implementing neighbor-net analysis with the variance of ordinary least squares.

We acquired 2.5 Gb of data, with 12,928,581 pairs of raw reads, resulting in 11,465,210 clean reads after filtering, averaging 208,458 ± 9,953 reads (mean ± standard error [SE]) per MIGseq sample. Post-alignment, sequence matrix size was 274,737 bp, with a total of 3,702 loci within 1,926 contigs. On average, we identified 1,564 ± 45 (range: 1,124–2,363) SNP loci. After filtering, 1,299 consensus SNP loci with <50% missing values for each locus from 55 accessions were used for analysis (Eaton and Overcast 2020, Evanno *et al.* 2005).

RADseq analysis yielded 16.1 Gb of data, with 187,617,514 clean reads after filtering and 3,411,228 ± 127,406 reads (mean ± SE) per sample. Following alignment, the sequence matrix size was 2,538,211 bp, encompassing 19,856 loci within 31,384 contigs. There were 12,569 ± 186 (range: 9,059–14,647) SNP loci identified. After filtering, 12,412 consensus SNP loci with <50% missing values for each locus from 55 accessions were used for the analysis.

Literature Cited

Eaton, D.A.R. and I. Overcast (2020) ipyrad: Interactive assembly and analysis of RADseq datasets. Bioinformatics 36: 2592–2594.

Evanno, G., S. Regnaut and J. Goudet (2005) Detecting the number of clusters of individuals using the software STRUCTURE: A simulation study. Mol Ecol 14: 2611–2620.
